# Microenvironment of* Mycobacterium smegmatis *Culture to Induce Cholesterol Consumption Does Cell Wall Remodeling and Enables the Formation of Granuloma-Like Structures

**DOI:** 10.1155/2019/1871239

**Published:** 2019-04-15

**Authors:** Ana Cristina Doria dos Santos, Victor Hugo de Souza Marinho, Pedro Henrique de Aviz Silva, Barbarella de Matos Macchi, Mara Silvia Pinheiro Arruda, Edilene Oliveira da Silva, José Luiz Martins do Nascimento, Chubert Bernardo Castro de Sena

**Affiliations:** ^1^Laboratory of Structural Biology, Institute of Biological Sciences, Federal University of Pará, Belém, PA, Brazil; ^2^Laboratory of Molecular and Cellular Neurochemistry, Institute of Biological Sciences, Federal University of Pará, Belém, PA, Brazil; ^3^National Institute of Science and Technology in Neuroimmunomodulation (INCT-NIM), Rio de Janeiro, RJ, Brazil; ^4^Laboratory of Extraction, Institute of Natural Science, Federal University of Pará, Belém, PA, Brazil; ^5^National Institute of Science and Technology in Structural Biology and Bioimaging, Rio de Janeiro, RJ, Brazil; ^6^Graduate Program in Pharmaceutical Science, Federal University of Amapá, Amapá, AP, Brazil

## Abstract

Pathogenic species of mycobacteria are known to use the host cholesterol during lung infection as an alternative source of carbon and energy. Mycobacteria culture in minimal medium (MM) has been used as an* in vitro* experimental model to study the consumption of exogenous cholesterol. Once in MM, different species of mycobacteria start to consume the cholesterol and initiate transcriptional and metabolic adaptations, upregulating the enzymes of the methylcitrate cycle (MCC) and accumulating a variety of primary metabolites that are known to be important substrates for cell wall biosynthesis. We hypothesized that stressful pressure of cultures in MM is able to induce critical adaptation for the bacteria which win the infection. To identify important modifications in the biosynthesis of the cell wall, we cultured the fast-growing and nonpathogenic* Mycobacterium smegmatis* in MM supplemented with or without glycerol and/or cholesterol. Different from the culture in complete medium Middlebrook 7H9 broth, the bacteria when cultured in MM decreased growth and changed in the accumulation of cell wall molecules. However, the supplementation of MM with glycerol and/or cholesterol recovered the accumulation of phosphatidylinositol mannosides (PIMs) and other phospholipids but maintained growth deceleration. The biosynthesis of lipomannan (LM) and of lipoarabinomannan (LAM) was significantly modulated after culture in MM, independently of glycerol and/or cholesterol supplementation, where LM size was decreased (LM_13-25KDa_) and LAM increased (LAM_37-100KDa_), when compared these molecules after bacteria culture in complete medium (LM_17-25KDa_ and LAM_37-50KDa_). These changes modified the cell surface hydrophobicity and susceptibility against H_2_O_2_. The infection of J774 macrophages with* M. smegmatis, *after culture in MM, induced the formation of granuloma-like structures, while supplementation with cholesterol induced the highest rate of formation of these structures. Taken together, our results identify critical changes in mycobacterial cell wall molecules after culture in MM that induces cholesterol accumulation, helping the mycobacteria to increase their capacity to form granuloma-like structures.

## 1. Introduction

Different species of mycobacteria are known to grow in a cholesterol-rich microenvironment to use the side chain of cholesterol to provide energy and carbon [1–5]. This utilization of cholesterol is known to be dependent upon nutrient availability, and* in vitro* experiments using bacteria cultures in minimal medium (MM) supplemented with cholesterol have been performed to induce a similar consumption of cholesterol by different species of mycobacteria [3, 6–8].

Additional studies have showed the metabolite profile obtained during the growth of different species of mycobacteria, comparing the bacteria's metabolism in complete Middlebrook 7H9 medium which have the glycerol as the defined carbon source, and MM supplemented with cholesterol [3, 8–10]. After cholesterol consumption, the mycobacteria are induced to start transcriptional and metabolic adaptations, in turn, upregulating the enzymes of the methylcitrate cycle (MCC). These organisms also accumulate a variety of primary metabolites that are known to be important substrates for cell wall biosynthesis, including methylsuccinate, 2-methylcitrate, mannose, mannose-1-phosphate, and trehalose 6-phosphate [8]. These data suggest that catabolism of cholesterol is a result of nutritional stress and may require the bacilli to make transcriptional and metabolic adaptations during intracellular growth. These adaptations may change many physiological conditions, especially pathways that are required to keep the bacilli alive.

The consumption of cholesterol is also been related to help pathogenic species of mycobacteria to survive during the infection of macrophages, suggesting an important physiological adaptation of the bacteria to win the phagocytosis [3, 8, 11, 12]. The infected host cells are then unable to kill the intracellular pathogen when the cholesterol is accumulated in cytosol, enabling bacteria to persist during the infection [1, 6, 7].

Intriguingly, when comparing different species of mycobacteria, fast-growing species, such as* Mycobacteria smegmatis*, which are usually nonpathogenic to humans and frequently used in laboratory investigations, can grow well in MM using cholesterol as a carbon resource [3, 4, 13]. As such,* M. smegmatis *is an attractive organism for modeling pathogenic species, such as* M. tuberculosis. *

The use of cholesterol is also related to the aggregation of infected macrophages to start to form granulomas, the hallmark of tuberculosis [14]. If the immune system fails to control the infection, the granuloma develops a core of cellular debris and lipids with thousands of viable bacilli from infected macrophages. This core is surrounded by macrophages that have accumulated lipid droplets (known as foamy cells), mononuclear leukocytes, and a fibrous sheath originating from the granuloma casemates [12, 14, 15]. Thus, cholesterol metabolism by bacteria and progression to active disease are interconnected and can occur for years after infection without an appropriate immune response, during which time, persistent bacteria grow into cholesterol-rich cells, forming the foamy macrophages [14, 16]. The bacilli within the phagosomes of foamy macrophages interact with lipid bodies, where the free bacteria survive, grow, and escape to the cytoplasm after the consumption of host cholesterol [15, 16].

The granuloma develops an inflammatory response that induces necrotic processes, killing the foamy macrophages and then releasing and accumulating bacilli and necrotic debris as caseum [14, 15]. In response to the infection, the host macrophages also initiate a transcriptional response against the bacilli and upregulate lipid metabolism to produce cholesterol to maintain the synthesis of inflammatory mediators, such as prostaglandin [17]. The accumulation of the host cholesterol also induces impressive bacterial growth within IFN-*γ*-activated macrophages, the primary macrophages involved in chronic tuberculosis [6].

Many metabolic pathways in mycobacteria are involved in fatty acid metabolism and help to define the distinctive architecture of the cell wall [8, 18, 19]. Unlike the cell membrane and peptidoglycan layers of other bacteria, the mycobacterial cell wall contains a large hydrophobic layer of mycolic acid, a long-chain *β*-hydroxyl fatty acid that is attached to the cell wall or to the trehalose dimycolate (TDM) layer. In addition to mycolic acid and TDM, the cell wall contains lipids and distinctive mannosylated glycolipids, such as phosphatidylinositol mannosides (PIMs), lipomannan (LM), and lipoarabinomannan (LAM). These compounds are known to be important for infection and bacteria survival in host cells due to the biological function of each molecule [20–23].

In this study, we used the fast-growing and nonpathogenic* M. smegmatis*, to investigate the effect of cholesterol consumption on cell wall biosynthesis and correlated our data with mechanisms of formation of granuloma-like structures.

## 2. Methods

### 2.1. Mycobacterial Strains and Culture Conditions

For each experiment,* Mycobacterium smegmatis* (Trevisan) of the Lehmann and Neumann strain (INCQS 00021, ATCC 607, Coleção de Microrganismos de Referência em Vigilância Sanitária-CMRVS, FIOCRUZ-INCQS, Rio de Janeiro, RJ) was initially grown on Middlebrook 7H10 agar plates (BD Biosciences) supplemented with 0.2% (w/v) glucose, 0.2% (v/v) glycerol, and 15 mM NaCl for three days. For the initial liquid culture, cells were grown in Middlebrook 7H9 broth (BD Biosciences) supplemented with 0.2% (w/v) glucose, 0.2% (v/v) glycerol, 15 mM NaCl, and 0.05% (v/v) tween 80 at 37°C in an orbital shaker at 150 r.p.m. for three days [24]. For defined carbon sources, cells at the stationary phase of growth, obtained from the initial liquid culture in Middlebrook 7H9 broth, were diluted to O.D._600_ 0.05 in fresh Middlebrook 7H9 broth, as before, or in minimal media containing asparagine (0.5 g/l), KH_2_PO_4_ (1.0 g/l), Na_2_HPO_4_ (2.5 g/l), NH_4_Fe(SO_4_)_2_  • 12H_2_O (50 mg/l), MgSO_4_  • 7H_2_O (0.5 g/l), CaCl_2_ (0.5 g/l), ZnSO_4_ (0.1 mg/l), 0.2% tyloxapol, vitamin B12 (10 mg/ml), and when appropriate, 0.1% glycerol and/or 0.01% cholesterol, as described previously [8]. We then worked with five groups: (1) 7H9+Gly; (2) MM+Gly; (3) MM+Gly+Chol; (4) MM+Chol; and (5) MM and growth was monitored by measuring optical density at 600 nm (*A*_600_) [24].

### 2.2. Filipin Staining of Cholesterol

Cultures of* M. smegmatis, *at the stationary phase of growth, were centrifuged (16,000* g* for 15 min at 4°C) and the cell pellets were washed three times with phosphate-buffered saline (PBS, pH 7.4), fixed with 3% paraformaldehyde for 1 h at room temperature, washed again, and incubated with glycine (1.5 g/ml) for 10 min at room temperature, to quench the paraformaldehyde. The mycobacteria cells were incubated with 0.05 mg/ml of filipin (Sigma-Aldrich) in the dark for 45 min at room temperature, as described previously [1]. To quantify cholesterol accumulation, the filipin-stained cells were analyzed by spectrofluorimetry (Victor X3, PerkinElmer; 340-nm excitation; and 380-nm emission).

### 2.3. Extraction and Analysis of PIMs, Phospholipids, GPLs, and TDM

To purify PIMs, phospholipids, GPLs, and TDM, cell pellets from 20 ml of Middlebrook 7H9 broth or MM cultures were sequential extracted in 20 volumes of chloroform-methanol (2:1, v/v), 10 volumes of chloroform-methanol (2:1, v/v), and 10 volumes of chloroform-methanol-water (1:2:0.8, v/v/v). Each extraction was followed by a 2 h incubation at room temperature. The combined organic solvents from each extraction were dried under a nitrogen stream before further purification in 1-butanol-water (1:1, v/v) biphasic partitioning. The 1-butanol phase was dried and applied to aluminum-backed thin-layer chromatography (TLC) silica gel 60 sheets (ALUGRAM). PIMs and phospholipids were analyzed in chloroform  : methanol  : 13 M ammonia  : 1 M ammonium acetate  : water (180:140:9:9:23, v/v/v/v/v) to further visualize PIMs using an orcinol-H_2_SO_4_ spray reagent and molybdenum blue spray reagent to visualize phospholipids [25]. GPL and TDM were analyzed in chloroform-methanol (9:1, v/v) and visualized using an orcinol-H_2_SO_4_ spray reagent, as described previously [26].

### 2.4. Extraction and Analysis of Mycolic Acids

The mycolic acids were extracted as described previously [27]. After lipid extraction, mycolic acids were released from cell pellets after incubation in methanol-toluene-sulfuric acid (30:15:1, v/v/v), for 12 h at 75°C. The mycolic acids from the extracted samples were further purified in 1 ml of hexane biphasic partitioning. The purified mycolic acid fraction was air dried at 45°C and then applied to aluminum-backed thin-layer chromatography (TLC) silica gel 60 sheets (ALUGRAM) using hexane-ethyl acetate (95:5, v/v). To visualize the mycolic acids, we used a vanillin/sulfuric acid spray reagent after baking at 150°C.

### 2.5. LM and LAM Extraction and Analysis

LM/LAM extraction was performed as described previously [24], using delipidated cell pellets resuspended in Tris-EDTA (pH 6.6)-saturated phenol-water (1:1, vol/vol) to extract for 2 h at 55°C. LM and LAM were separated by electrophoresis in 4-20% Tris-Tricine SDS-PAGE (Bio-Rad) and visualized after staining the glycans [28]. The image of the gel with the stained LM and LAM was then digitalized and analyzed by densitometry using ImageJ 1.48v software.

### 2.6. Hydrophobicity Analysis

Cellular hydrophobicity was analyzed by hexadecane partitioning, as described previously with modifications [25, 29]. Cultures of* M. smegmatis* were grown to, approximately, the early stationary phase. Cell culture volumes equivalent to 0.5 OD_600_ were pelleted, washed once in PBS, and resuspended in 750 *μ*l of PBS. The resuspended cells were mixed with 25 *μ*l of hexadecane for 2 min, and the two phases were then allowed to separate. After 15 min, 150 *μ*l aliquots of the lower aqueous phase were transferred to a 96-well plate, and the* A*_600_ was measured. The cell surface was calculated using the following formula: % hydrophobicity = [(*A*_600_ before mixing –* A*_600_ after mixing)/*A*_600_ before mixing]x100.

### 2.7. Susceptibility Assay against H_2_O_2_

*M. smegmatis *were grown in Middlebrook 7H9 and MM, as described before, until the early stationary phase. The bacteria were pelleted (1000* g* for 15 min at 4°C) and recovered in 5 ml PBS (pH 7.2) with 20 mM H_2_O_2_ to reach OD_600_ at 0.1. Bacteria were then treated for 3 h at 37°C [30]. After the treatment, aliquots of 25 *μ*l were spotted onto Middlebrook 7H10 and incubated for 3 days to identify bacteria resistant [26].

### 2.8. Macrophage Infection and Granuloma-Like Structure Analysis

The culture and infection of J774.1 cells, a mouse macrophage cell line, were performed as described previously with some modifications [31]. J774.1 cells were seed onto 24-well tissue culture plates with sterile coverslips at 0.5 x 10^5^ cells per well and incubated for 2 days until reaching 90% confluency. The cells were infected with* M. smegmatis *at a multiplicity of infection (MOI) of 100:1, using bacteria cultures in Middlebrook 7H9 and MM at early stationary phase, as described before. After 1 h of macrophage infection, gentamycin (10 *μ*g ml^−1^) was added to kill all extracellular bacteria. After 12 h after infection (h.p.i.), the supernatants were removed, and the infected cells were heat-fixed and stained using the Ziehl-Neelsen method, as described previously [31]. The samples were analyzed using an Axio Zeiss fluorescent microscopy (Zeiss) equipped with an AxioCam MTC digital camera system (Zeiss) and Zen software (Zeiss).

### 2.9. Statistical Analysis

All experiments were repeated twice for each sample group. When necessary, the data are expressed as the means ± standard deviation (SD) of biological triplicates. Significant differences were accessed by one-way ANOVA followed by* post hoc *Turkey's test, using Biostat 5.0 software.* p < 0.05 *was considered statistically significant.

## 3. Results

### 3.1. Cholesterol Does Not Recuperate Bacterial Growth Rate in Minimal Medium

**Figure 1:**
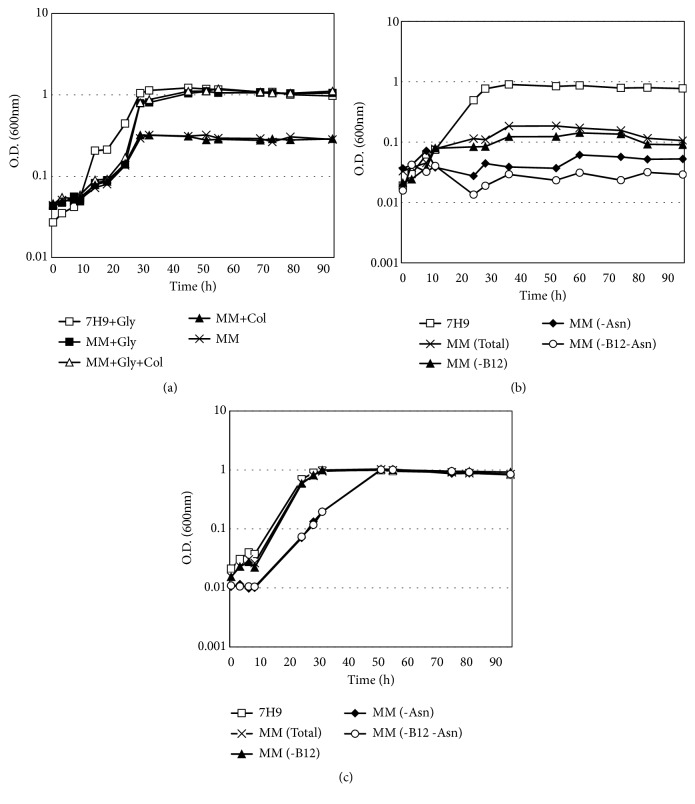
*Mycobacterium smegmatis* modifies its growth rate in minimal medium. Growth rate (*A*_600  nm_) in Middlebrook 7H9 broth and (a) minimal medium (MM) with or without glycerol and/or cholesterol supplementation or (b) only in MM with or without B12 vitamin (B12) and/or asparagine (Asn). Viable cells after culture in MM with or without B12 and/or Asn were identified after reculturing each group in complete Middlebrook 7H9 broth (c).

To investigate the ability of mycobacteria to grow in MM and to induce the use of cholesterol, we first compared the growth rate of* M. smegmatis* in complete medium Middlebrook 7H9 broth, which is normally supplemented with glycerol, with cultures in MM, with or without glycerol and/or cholesterol supplementation. Nutrient depletion resulted in the slow growth of mycobacteria; however, glycerol supplementation, with or without cholesterol (MM+Gly and MM+Gly+Chol groups), was sufficient for cells cultured in Middlebrook 7H9 broth to achieve an optical density at 600 nm (O.D._600;_ 7H9+Gly group with O.D._600_ of 1.04 - 1.11 at stationary phase) equivalent to that observed under usual mycobacterial culture conditions (Figure 1(a)). In contrast, bacteria cultured in MM without glycerol (MM+Chol and MM groups) did not reach the same stationary phase density as those of the other groups, maintaining a stationary phase at O.D._600_ of 0.29-0.32, but this result is likely due to low concentration of cholesterol, which is limited by solubility. To investigate which compound of MM is the main source to maintain the cell growing without glycerol and cholesterol as carbon source, the growth rate was compared between complete medium Middlebrook 7H9 and MM with/without vitamin B12 and asparagine (Asn) (Figure 1(b)). These conditions of cell cultures did not show growth only when the asparagine is missing (-Asn and -B12-Asn). This result indicates that the asparagine is the main compound in MM to maintain the mycobacteria growing without supplementation of glycerol and/or cholesterol. Taking together the results of Figures 1(a) and 1(b), we also need to consider that the irrelevant bacteria growth in MM without glycerol can be due the asparagine and not because the low concentration of cholesterol, as showed in MM+Chol group. To analyze cell viability after culture in MM with/without vitamin B12 and asparagine, cells in stationary phase from each group were reinoculated (O.D_600nm_ 0.01) in complete medium Middlebrook 7H9 broth and the cell growth was monitored. As showed in Figure 1(c), the cells from culture in MM without asparagine slowed the growth but all groups reached the growth rate up to the cell culture of complete medium Middlebrook 7H9, indicating partial cell death without asparagine in MM.

### 3.2. Mycobacterium smegmatis Makes Use of Cholesterol Only When Glycerol Is Not Available in Minimal Medium

**Figure 2:**
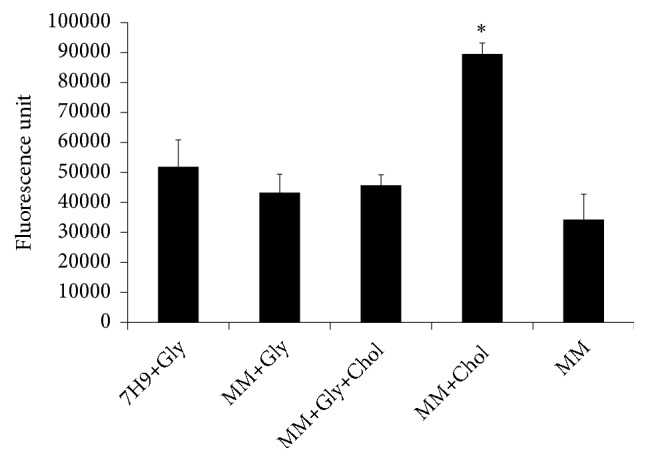
Minimal medium inducing* Mycobacterium smegmatis* to use cholesterol. Bacteria cells in the early stationary phase of growth in Middlebrook 7H9 broth or minimal medium (MM), with or without glycerol and/or cholesterol, were assayed by spectrofluorimetry after filipin staining. Culture groups: Middlebrook 7H9 broth (7H9+Gly) and minimal medium (MM) with or without glycerol and/or cholesterol (MM+Gly; MM+Gly+Chol; MM+Chol; and MM). *∗*p < 0.05 compared to 7H9+Gly group. This experiment was performed in triplicate, and the values are plotted as means ± SD.

Spectrofluorimetric analysis of filipin-stained cells was used to quantify the presence of cholesterol after growth of mycobacteria. Bacteria cultured in MM presented a statistically significant presence of cholesterol, compared with the bacteria cultured in Middlebrook 7H9 medium, only when the supplementation of cholesterol was administered in the absence of glycerol (Figure 2, MM+Chol). These observations indicate that mycobacteria prefer glycerol to cholesterol for use as its main carbon and energy source.

### 3.3. Alterations in Cell Wall Lipid Profiles

**Figure 3:**
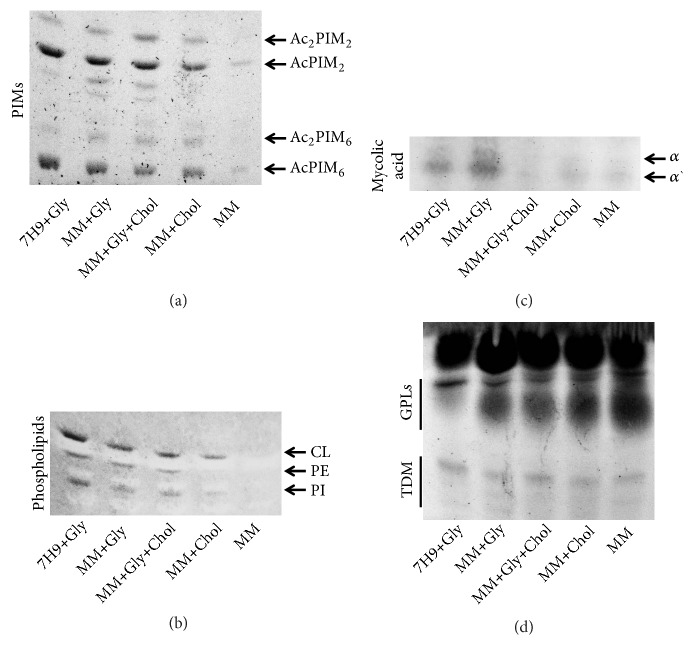
The cell wall lipid profile is altered after culture in minimal medium. Lipid fractions of the mycobacterial cell wall were extracted from bacteria in stationary phase cultures, grown in Middlebrook 7H9 broth or minimal medium (MM) with or without glycerol and/or cholesterol supplementation (7H9+Gly, MM+Gly, MM+Gly+Chol, MM+Chol, and MM, respectively), by thin-layer chromatography (TLC) using solvent systems. The fractions were then stained for the following components: (a) glycolipids, using the orcinol staining reagent to identify PIMs; (b) phospholipids, using the molybdenum blue staining reagent to identify cardiolipin (CL), phosphatidylethanolamine (PE), and phosphatidylinositol (PI); (c) mycolic acids using chromic acid reagent; and (d) glycopeptidolipid (GPL) and dimycolate trehalose (TDM) using the orcinol staining reagent. The data are representative results of three independent experiments, showing only the relevant segments of the TLC plates.

To investigate whether bacteria cultured in MM presented changes in the cell wall molecules, we examined lipid fractions of cell wall extractions. The presence of PIMs (Figure 3(a)) and phospholipids (phosphatidylinositol-PI, cardiolipin-CL, and phosphatidylethanolamine-PE, Figure 3(b)) was drastically affected only when glycerol and cholesterol were not available in the MM cultures. These data suggest that glycerol and/or cholesterol are important for maintaining small lipids on the mycobacteria surface during their growth in MM. In contrast to observations for PIMs and phospholipids, the presence of mycolic acid was affected during growth in MM when the cholesterol was used or lacking, independently of cosupplementation with glycerol (MM+Gly+Chol, MM+Chol and MM of Figure 3(c)). Furthermore, when the culture in MM was supplemented only with glycerol, the cells showed a large presence of mycolic acid (MM+Gly of Figure 3(c)).

As shown in Figure 3(d), the TDM did not show any alteration in its presence in cell wall of any culture groups, but surprisingly the GPLs were highly present after growth in MM, independently of supplementation with or without glycerol and/or cholesterol. These data indicate that culture in MM induces bacteria to use mycolic acid to maintain the level of TDM, thus decreasing the accumulation of mycolates on cell wall.

Intriguingly, the high presence of GPLs during culture in MM, independently of glycerol/cholesterol supplementation (upper panel of Figure 3(d)), suggests that changes are a response to the new environment. These changes may be necessary to maintain cell wall integrity after the reduction in mycolic acid and to prepare the cells to ensure survival during growth, independently of the presence and further consumption of cholesterol.

### 3.4. Minimal Medium Modulates the Length of LAM and LM

**Figure 4:**
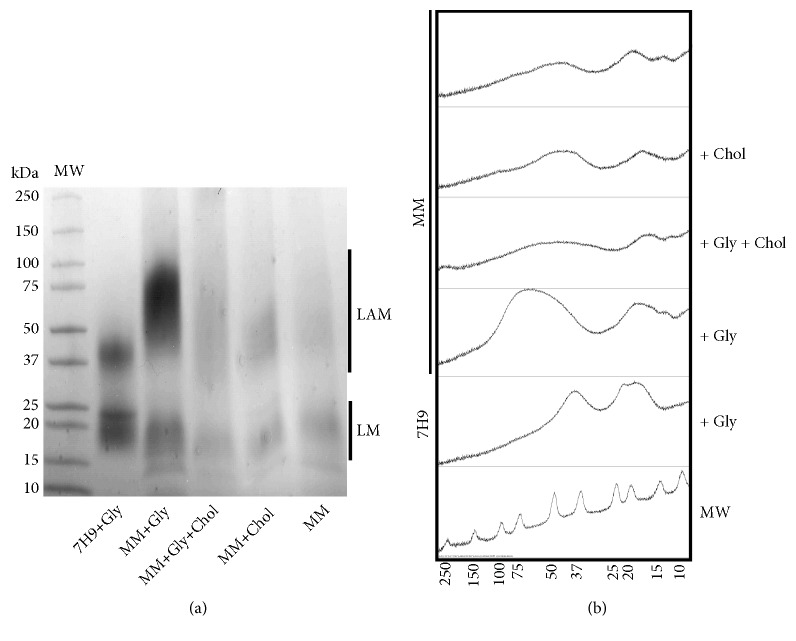
The lengths of LM and LAM are modulated during growth in minimal medium. (a) Glycolipid fractions of the mycobacterial cell wall of* M. smegmatis, *in the stationary phase of growth in Middlebrook 7H9 broth (7H9+Gly) or minimal medium (MM) with or without glycerol and/or cholesterol supplementation (7H9+Gly, MM+Gly, MM+Gly+Chol, MM+Chol, and MM groups), were separated by electrophoresis in 4-20% Tris-Tricine SDS-PAGE and stained for glycans to visualize LM and LAM. (b) Densitometric analysis of stained LM and LAM in 4-20% Tris-Tricine SDS-PAGE was performed using the ImageJ 1.48v software. The staining of LM and LAM is a representative result of three independent experiments, showing only the relevant segment of the SDS-PAGE. MW: molecular weight standard.

To determine whether the cultures in MM are able to modulate the length of LM and LAM, the glycoconjugate fraction was examined by SDS-PAGE followed proteoglycan staining. Our data show that the different culture conditions, even with or without cholesterol and/or glycerol supplementation, resulted in the biosynthesis of unusual LM and LAM in* M. smegmatis. *LM became smaller and LAM became bigger, compared to these molecules when isolated from cells cultured in Middlebrook 7H9 broth (Figure 4(a)). These alterations were much more evident in the LAM molecules extracted from* M. smegmatis *after culture in MM supplemented with glycerol (MM+Gly and MM+Gly+Chol); however, like the result of mycolic acid, the presence of LM and LAM was changed during growth in MM, showing the largest presence of these molecules when the culture in MM was supplemented only with glycerol (MM+Gly), but the presence was affected during growth in MM when the cholesterol was used or lacking, independently of cosupplementation with glycerol (MM+Gly+Chol, MM+Chol, and MM).

To estimate the size of LM and LAM, we used densitometry to analyze each sample after Tris-Tricine SDS-PAGE and glycoconjugate staining. As shown in Figure 4(b), the unusual LM appears with a molecular weight of 13-25 KDa (LM_13-25_) in all groups after cultures in MM (MM+Gly, MM+Gly+Chol, MM+Chol, and MM groups); this LM is smaller than that of the control group of bacteria cultured in Middlebrook 7H9 broth (7H9+Gly), which presented a molecular weight of 17-25 KDa (LM_17-25_). In regard to LAM, LAM from bacteria cultures in MM (MM+Gly, MM+Gly+Chol, MM+Chol, and MM groups) presented a molecular weight of 37-100 KDa (LAM_37-100_), compared to the LAM of 37-50 KDa (LAM_37-50_) extracted from bacteria cultured in Middlebrook 7H9 broth (7H9+Gly).

Our results suggest that LM and LAM are able to change their length when bacterial growth occurs in environments that differ from those of the Middlebrook 7H9 broth (the customary nutrient culture condition for mycobacteria). Data also suggest that when glycerol or cholesterol is the main carbon and energy sources in MM cultures, the mycobacteria are able to modulate the glycolipid length and accumulate unusual LM and LAM.

### 3.5. Minimal Medium Changes Bacterial Cell Wall Hydrophobicity and H_2_O_2_ Susceptibility

**Figure 5:**
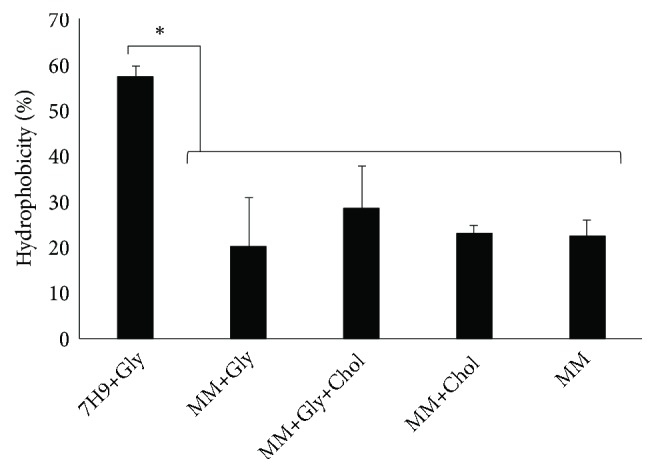
Growth of* M. smegmatis *in minimal medium changes the cell surface hydrophobicity. Hexadecane partitioning of* M. smegmatis* in stationary phase cultures in Middlebrook 7H9 broth (7H9+Gly) or minimal medium (MM) supplemented with glycerol and/or cholesterol as the defined carbon and energy source (MM+Gly, MM+Gly+Chol, and MM+Chol). This experiment was performed in triplicate, and the values are plotted as means ± SD. Tukey's test was performed to compare the 7H9+Gly with other groups (*∗*, p<0.01).

To evaluate whether cultures in MM present modifications in cell surface hydrophobicity, we assayed the partitioning of bacteria into the hexadecane. The bacteria from cultures in MM were more likely to partition into the hexadecane phase than the cells grown in Middlebrook 7H9 broth, independently of supplementation with glycerol and/or cholesterol (Figure 5). This result indicates that metabolic adaptation in the new environment makes the cell wall surface less hydrophobic, which may change the bacterial response to host immune response.

**Figure 6:**
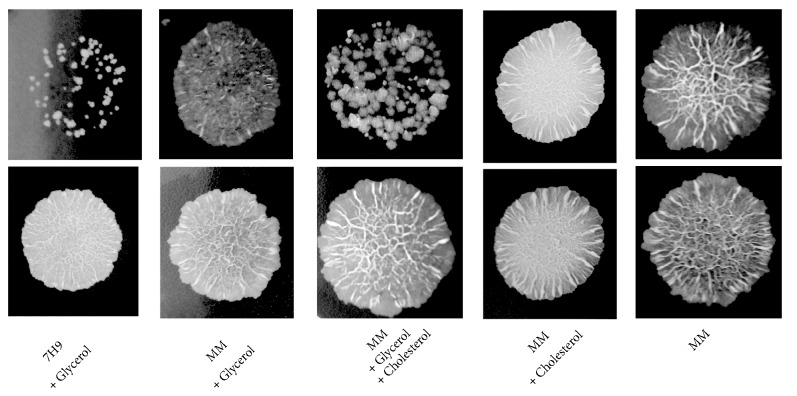
Sensitivity of bacteria to H_2_O_2_.* M. smegmatis* in the stationary phase that were cultured in Middlebrook 7H9 broth or minimal medium (MM), with or without glycerol and/or cholesterol, were treated with 20 mM H_2_O_2_ for 3 h and then spotted onto solid Middlebrook 7H10 broth. The images show cultures of the spotted bacteria that are representative of three independent experiments.

Due to the changes observed in the mycobacterial cell wall after growth in MM, we analyzed the bacteria susceptibility to H_2_O_2_. As shown in Figure 6, the analysis of susceptibility to H_2_O_2_ showed that the bacteria grown in MM, independently of supplementation with glycerol and/or cholesterol, showed resistance to 20 mM H_2_O_2_ treatment, in contrast to cells cultured in Middlebrook 7H9 broth, which were easily killed (Figure 6).

### 3.6. Cholesterol Consumption Increases the Formation of Granuloma-Like Structures

**Figure 7:**
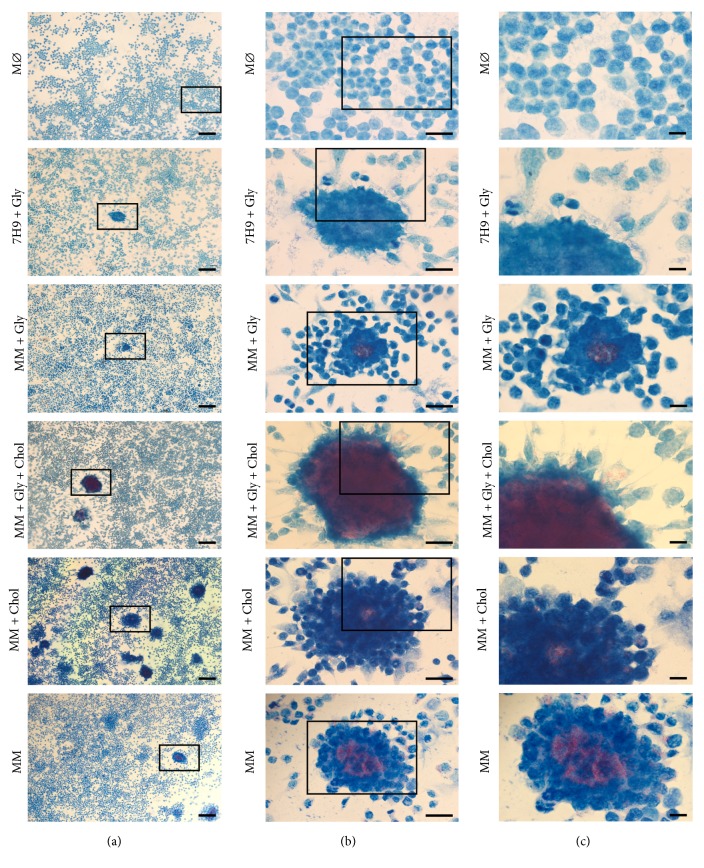
Formation of granuloma-like structures. Images of light microscopy and Ziehl-Neelsen staining showing macrophage aggregates of infected cells. The formations of granuloma-like structures occurred after the infection of macrophage cells with* M. smegmatis* after their culture in 7H9 Middlebrook supplemented with glycerol (7H9+Gly) or in MM, with or without glycerol and/or cholesterol supplementation (MM+Gly, MM+Gly+Chol, MM+Chol, and MM), compared with noninfected cells (M*ϕ*, control group). The images in low magnification ((a) bar size 200 *μ*m) were amplified ((b) and (c) bar sizes 50 and 20 *μ*m, respectively) to analyze cell morphology and the formation of granuloma-like structures by clusters of macrophages (in blue) surrounding clumps of bacilli (in red). The images are representative of three independent experiments.

J774.1 cells were infected with mycobacteria and cultures were then stained with Ziehl-Neelsen at 12 h.p.i. for observation of the reorganization of macrophage and the formation of granuloma-like structures formed by clusters of macrophages surrounding clumps of bacilli (Figure 7). The use of cholesterol by* M. smegmatis *induced bigger granuloma-like structures with the intense migration of activated and vacuolated macrophages and intracellular bacteria (Figure 7, groups MM+Gly+Chol and MM+Chol).

**Figure 8:**
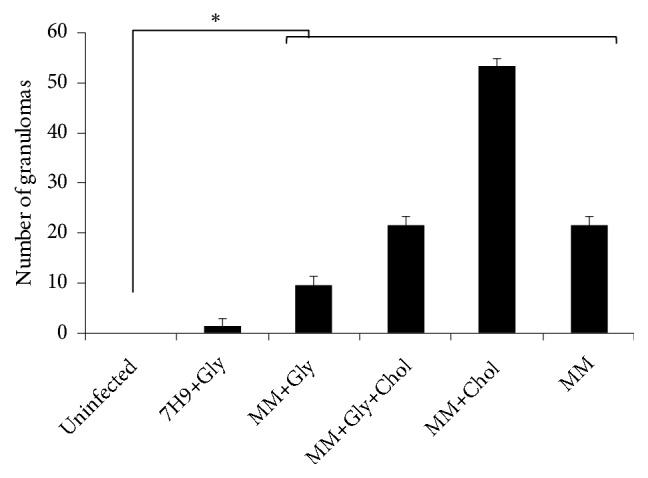
Number of granuloma-like structures. The granuloma-like structures in infected macrophages were quantified after Ziehl-Neelsen staining and the number of granulomas was plotted as mean ± SE. Data from noninfected macrophages (control) were compared with macrophages that were infected with* M. smegmatis* after their culture in Middlebrook 7H9 broth (7H9+Gly) or minimal medium (MM), with or without glycerol and/or cholesterol (MM+Gly; MM+Gly+Chol; MM+Chol; and MM). *∗*p < 0.05 compared with the uninfected control group. The experiment was performed in triplicate.

When comparing all groups of infection with the control group (noninfection), all of the groups containing macrophages infected with* M. smegmatis *after culture in MM, independently of supplementation, showed significant increases in granuloma-like structures (Figure 8). However, the infection with mycobacteria after culture in MM, supplemented with only cholesterol, demonstrated the highest number of granuloma-like structures, when compared with the other groups of infected macrophages (Figure 8, MM+Chol group).

## 4. Discussion

The results of the present study provide the first evidence that* M. smegmatis* cultures in MM favor a microenvironment that induces not only cholesterol consumption, but also changes in the structure of the bacterial cell wall and cell surface hydrophobicity. These changes induced bacterial resistance to H_2_O_2_ and increased the efficiency of formation of granuloma-like structures after infection of macrophage by this nonpathogenic bacillus cultured in MM previously. Other studies have demonstrated that cholesterol is a carbon source that is critical for sustaining the metabolism of mycobacteria during infection [6, 8].

The* in vitro* induction of cholesterol consumption by mycobacteria has been observed during culture in MM, a modified Sauton's media, which is known to slow the growth of mycobacteria [1, 6, 8, 25]. This behavior was confirmed when we analyzed the presence of cholesterol and bacteria growth of* M. smegmatis* cultures in MM with or without cholesterol and/or glycerol, comparing with culture in complete medium Middlebrook 7H9 broth, which is conventionally supplemented with glycerol. We observed that cultures in MM induced the use of cholesterol only when the glycerol was lacking, but it was not efficient for recuperating the growth rate of* M. smegmatis*. The fast-growing and nonpathogenic* M. smegmatis* is known to be able to consume cholesterol from the growth media throughout their growth cycle [32]. Culture of pathogenic* M. tuberculosis *and* Mycobacterium bovis *has been reported to modify the bacteria growth after changing the concentration of cholesterol [6, 33]. It is known that the cell density with cholesterol supplementation is lower than glycerol and it is due to low concentration of cholesterol during cultures, what is limited by solubility of cholesterol [6]. Besides, different pathogenic species of mycobacteria from their experimentally infected animals prefer to incorporate carbons from lipids than from glycerol to use these carbons for lipid biosynthesis, indicating that the preference for different carbon sources is depending of metabolic bacteria condition [34].

Is early known that the saprophytic* M. smegmatis* can utilize the amino acids to support metabolism but the* M. tuberculosis *is also known to transport, secrete and hydrolyze the asparagine to release the ammonia to become resistant against the acid stress during the culture in MM, mimetizing* in vitro *the limited microenvironment of the infection [35, 36]. It can explain our results about the growth of* M. smegmatis* in MM without any glycerol and/or cholesterol supplementation. Thus, the induction of asparagine and cholesterol consumption during limited nutrient conditions, like culture in MM, are straightly related to physiology and virulence of mycobacteria cells.

The cultures of pathogenic and nonpathogenic mycobacteria in MM do the upregulation of genes that uptake and degrade lipids, what is known to occur when cholesterol is used as the main carbon source in limited nutrient culture condition [3, 5, 37]. These metabolic alterations observed during cholesterol catabolism centered on propionyl-CoA and pyruvate pools, substrates of the methylcitrate cycle, and the glyoxylate cycle, inducing the accumulation of several metabolites for different pathways including the cell wall biosynthesis [8].

The lipid analysis of the mycobacteria cell wall revealed that PIMs and phospholipids are impaired only when MM cultures were not supplemented with glycerol and cholesterol. However, mycolic acids and GPLs were the major cell wall glycolipids and presented changes in their accumulation after culture in MM. When comparing the lipids of the mycobacteria cell wall after culture in complete Middlebrook 7H9 broth or MM, the accumulation of mycolic acid was impaired when cultures in MM were supplemented with or without glycerol and/or cholesterol supplementation. Conversely, the accumulation of mycolic acid was surprisingly high when MM was supplemented only with glycerol. These results are in agreement with the long-term survival of mycobacteria during starvation conditions that require recycling of mycolic acids to generate carbon and energy [38]. The mycolic acids, which are long-chain *α*-alkyl *β*-hydroxy fatty acids, play a crucial role in the cell wall architecture, impermeability, and natural antibiotic resistance of mycobacteria [39, 40]. It is known that* mce1 *operon, which resembles ATP-binding cassette (ABC) transporters for lipid importation, including cholesterol, plays important role to control the accumulation of free mycolic acid in the cell wall and formation of lung granulomas [41, 42]. Because the mycolates are attached to the arabinogalactan layer and also be a component of the trehalose, we analyzed the GPL and TDM.

In contrast with mycolic acid, the GPLs presented a surprisingly high accumulation in all groups of cells cultured in MM, independently of the presence or absence of supplementation. In contrast to observations for mycolic acids and GPLs, the TDM did not present a reduction in accumulation during cultures in MM. Thus, our data show that mycobacteria make important adjustments in cell wall architecture after the use of cholesterol, allowing them to grow in MM. GPLs, together with mycolic acids, is also the major constituent of the outer part of the cell wall and contribute to cell wall structural integrity [18, 43]. Both molecules are important during macrophage infection as they protect the bacilli against oxidative stress; furthermore the GPLs interact with the phagosomal/phagolysosomal compartment and induce immunosuppression during infection [18, 43]. This reflects the physiological adaptation of mycobacteria during growth that enables them to use cholesterol as a carbon source, as observed during infection [6]. Data, therefore, suggest that pathogenic mycobacteria are lipolytic during* in vivo *infection and need fatty acid catabolic pathways to survive inside the host.

The analysis of LM and LAM in mycobacteria showed that the lengths of these molecules were different in the cells cultured in complete medium (LM_17-25_ and LAM_37-50_), compared to those cultured in MM (LM_13-15_ and LAM_37-100_), independently of the presence or absence of glycerol and/or cholesterol. These findings suggest that the MM can induce significant changes in LM and LAM biosynthesis. Consistent with this interpretation, growth of* M. tuberculosis *in MM to induce the consumption of cholesterol is known to induce the bacteria to accumulate mannose and mannose-1-phosphate [8], which are important substrates for the synthesis of PIMs, LM and LAM by mannosyltransferases. The activities of mannosyltransferases are known to control mannose elongation and the lengths of LM and LAM [24], compromising the cell wall integrity [26]. Another interesting relation with this result is that is known that pathogenic species of mycobacteria synthesize LAM with a larger average molecule weight, in contrast to observations in nonpathogenic species [44]. Like the mycolic acid contents in cell wall, the quantity of LM and LAM was also reduced after cultures in MM supplemented with or without glycerol and/or cholesterol but high when MM was supplemented only with glycerol, comparing these contents after culture in complete Middlebrook 7H9 broth. These founds suggest a relationship of mycolic acid-arabinogalactan-peptidoglycan layer with LM and LAM to maintain the integrity of the* M. smegmatis *cell wall.

The catabolism of cholesterol by mycobacteria requires sterol degradation (ring degradation) and side chain *β*-oxidation to produce pyruvate and acetyl-CoA or propionyl-CoA, known to be the primary metabolites of the glyoxylate cycle and methylcitrate cycle, respectively [10]. Pyruvate might also be used for glycolysis/gluconeogenesis to maintain the amount of sugar in the bacteria, including the mannose, the substrate of mannosyltransferase for LM and LAM biosynthesis [5, 8]. It is known that branching and elongating mannosyltransferases are controlling the elongation of mannose backbone of LM and LAM, changing the sized and quantities of these molecules in cell wall [24]. Taken together with our data, the growth of the nonpathogenic* M. smegmatis *in MM suggests that the accumulation of mannosyltransferase substrates during cultures in MM could change the activity of these enzymes and, thus, modify the elongation and quantities of LM and LAM, preserving the biosynthesis of PIMs. These data regarding the mycobacteria cell wall are in agreement with our analysis of cell surface hydrophobicity, which changed after culture in MM, independently of glycerol or cholesterol supplementation.

Another important finding of this study was that changes in the bacterial cell wall after culture in MM showed bacteria resistant against hydrogen peroxide attack, suggesting that the new environment in MM might change the physiology of mycobacteria to allow the bacilli to become resist to phagocytosis. The infection of J774 macrophage cells revealed that the changes in the bacterial cell wall, after the culture of these bacteria in MM, induced the infected macrophages to present a different behavior and, consequently, resulted in cellular reorganization to form granuloma-like structures. Notably, the quantification of the granuloma-like structures showed that the use of cholesterol by the nonpathogenic* M. smegmatis *was significantly augmented. It has been reported that clinical strains of* M. tuberculosis*, which are known to use the cholesterol as carbon and energy source, show different virulence and immunogenicity, changing the regulation of host genes involved in cholesterol metabolism, proinflammatory cytokine response, prostaglandin synthesis, and T cell activation during* in vitro *and* in vivo *infection [17]. Taken together our results suggest that the nonpathogenic* M. smegmatis* after culture in MM to induce the use of cholesterol plays additional role during macrophage infection, showing a different host cell response. It might favor more stimuli from infected host cells to increase cellular migration and the formation of granuloma-like structures. These findings might provide clues regarding the persistence of mycobacteria during chronic infection, wherein the use of host lipids, especially cholesterol, as alternative sources of carbon and energy, may allow the bacilli to survive within macrophages.

## 5. Conclusion

Thus, our data demonstrate that microenvironment during culture in MM does not only induce the use of cholesterol but also make critical changes in the cell wall of nonpathogenic* M. smegmatis*. The changes help these mycobacteria to modify the cell surface hydrophobicity and then become resistant against hydrogen peroxide what facilitate the immunomodulation of macrophage infected cells, increasing the formation of granuloma-like structures, the hallmark of tuberculosis. These data show how the mycobacteria can be adapted to new environment to become able to win the infection and produces the disease.

## Data Availability

The data used to support the findings of this study are available from the corresponding author upon request.
